# Using Scopus and OpenAlex APIs to retrieve bibliographic data for evidence synthesis. A procedure based on Bash and SQL

**DOI:** 10.1016/j.mex.2024.102601

**Published:** 2024-02-03

**Authors:** Robin Harder

**Affiliations:** Environmental Engineering Group, Department of Energy and Technology, Swedish University of Agricultural Sciences (SLU), Uppsala, Sweden

**Keywords:** Systematic map, Systematic review, Citation map, Bibliographic analysis, Bibliometric analysis, API-CODEBASE

## Abstract

Evidence synthesis methodologies rely on bibliographic data. The process of searching and retrieving bibliographic data can be supported by using bibliographic APIs. This paper presents a collection of code that serves both as a recipe book and a finished working example of how to interact with Scopus and OpenAlex APIs for the purpose of supporting evidence synthesis. While the procedure and code base presented here were developed as part of an evidence synthesis project in the field of nutrient recovery from human excreta and domestic wastewater for reuse in agriculture, the procedure and code base should be useful more broadly for evidence syntheses or bibliographic analyses also in other fields.

• This paper presents a working example of how to interact with Scopus and OpenAlex APIs
• The code base is written in SQL (MySQL) and Unix Shell (Bash)
• The procedure was developed in an MacOS environment but should be portable to other environments

## Graphical abstract

**Figure:**
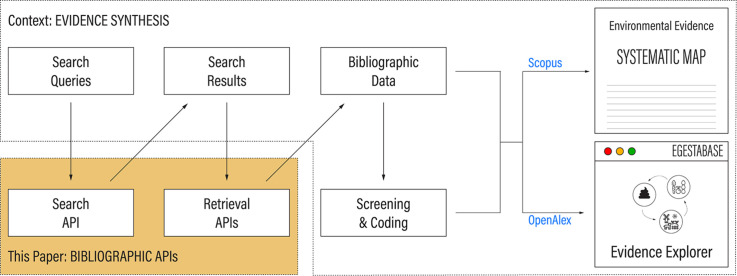

Specifications Table

**Table:** 

Subject area:	Environmental Science
More specific subject area:	*Evidence Synthesis*
Name of your method:	API-CODEBASE
Name and reference of original method:	*Not applicable*
Resource availability:	doi:10.17632/b4j39ccj8t.1

## Method details

 

## Introduction

Global research output is rapidly growing year after year. Bibliometric analysis methodologies, such as citation analysis, help assess the development of the scientific literature in a given research field by understanding the inter-relationships and impacts of publications, authors, institutions, countries, and journals [3]. Evidence synthesis methodologies, such as systematic maps and reviews, aim at collating, describing and summarizing relevant research on a specific topic or research question [8]. Evidence synthesis can be supported by machine learning algorithms, such as topic modelling, to provide substantial enhancement to the productivity of evidence synthesis [4,5,10,14].

All of above research methodologies rely on bibliographic data from sources such as Scopus [1], Web of Science [2], Dimensions [7], Crossref [6], or Microsoft Academic Graph [17]. All of these sources of bibliographic data vary in comprehensiveness, selectivity, and overlap [15]. A relatively new data source is OpenAlex, which was launched in January 2022, and is considered a replacement for Microsoft Academic Graph, which was retired in December 2021 [11,13].

Retrieval of bibliographic data from bibliographic data sources can typically take place through a website. When handling searches that yield several thousands of records, however, data export from these websites can become rather cumbersome. This is because the number of records that can be exported at once is typically limited, thus requiring repetitive manual exporting of a few hundred or thousand records at a time. Moreover, the website export functionality does not necessarily cover all bibliographic data that is potentially available from the respective source.

As an alternative to manual data retrieval through a website, application programming interfaces (APIs) provide a way to retrieve bibliographic data using a customized computer algorithm that directly communicates with the respective data source. Bibliographic APIs are available for a large number of bibliographic data sources and provide a standardized way of interacting with these sources. While good documentation is typically available from the respective API provider, custom algorithms are still required to extract data elements from the retrieved records in a way that is purposeful for a given research goal (e.g., [16]). In this regard, the Scholarly API Cookbook by the University of Alabama Libraries [12] provides a valuable and comprehensive collection of code examples (i.e., recipes) that demonstrate how to work with various scholarly APIs.

In this paper, a collection of working code is provided that was developed in the context of the project ‘End-of-wastewater’ – this project aimed at: (1) collating and summarizing scientific research on technologies that facilitate the recovery and reuse of plant nutrients and organic matter found in human excreta and domestic wastewater; and (2) to present this evidence on an online evidence platform in a way that can be navigated easily [9]. Unfortunately, the Scholarly API Cookbook was discovered only after finalizing the code base presented here. Otherwise, certain aspects might have been implemented somewhat differently. Either way, the procedure and code base shared here provide a working example for interacting with scholarly APIs that should be useful more broadly than the evidence synthesis project within which it was developed. In that sense, it can serve as both a recipe book and an example of a finished dish.

## Basic choices and preparations

Literature reviews can be facilitated by tools such as EPPI-Reviewer. EPPI-Reviewer is a web-based software for research synthesis that provides broad functionality and generally is very useful for screening and coding records. As our dataset in the project 'End-of-wastewater' grew to over 150 000 records, however, we experienced two major challenges: (1) screening and coding could not be performed with a sufficiently high speed; and (2) import and export of records and screening and coding results to and from EPPI-Reviewer became rather impractical. For these reasons, we started to develop our own bespoke web-based tool for rapid screening and coding. It is in this context that we also needed procedures to interact with scholarly APIs.

The procedure presented in this paper is underpinned by three basic choices: (1) the scholarly APIs to tap into, (2) the programming language used to interact with the chosen APIs, and (3) the database management system used to handle the retrieved bibliographic data.

### Scholarly API

While some APIs are openly accessible and do not require special authentication (e.g., Crossref, OpenAlex), other APIs require an affiliation with a subscribing institution, and a registration for an API key to use for authentication in API queries (e.g. Scopus, Web of Science). Before choosing and interacting with a scholarly API, it is important to review not only access and functionality, but also usage policies regarding aspects such as use cases, query limits, and data reuse policies. Table 1 shows the outcome with regard to our evidence synthesis in the project ‘End-of-wastewater’.

**Table 1:** Review of access, functionality and usage policies for data sources considered in the ‘End-of-wastewater’ project.

Data Source	Specific APIs	Access	Functionality	Usage Policies
Scopus	Abstract Retrieval API Author Retrieval API Affiliation Retrieval API	Subscription (Available) Subscription (Available) Subscription (Available)	Sufficient Sufficient Sufficient	Partly Fulfilled Partly Fulfilled Partly Fulfilled
Web of Science	API Lite API Expanded	Free Subscription (Not Available)	Limited Limited	Not Checked Not Checked
Crossref	REST API	Free	Limited	Not Checked
OpenAlex	Works API Authors API Institutions API Sources API	Free Free Free Free	Sufficient Sufficient Sufficient Sufficient	Fulfilled Fulfilled Fulfilled Fulfilled

Based on Table 1, Scopus and OpenAlex were chosen: Scopus as the preferred data source for the systematic map (as OpenAlex was still experimental at the time the systematic map was compiled), and OpenAlex as the preferred data source for the online evidence platform (as this use case is not allowed by the Scopus APIs, and OpenAlex has since moved beyond experimental). To access the Scopus APIs, one is required to register and get an API key at https://dev.elsevier.com/.

Note that, while the use of these two data sources was deemed sufficient in the project 'End-of-Wastewater', other evidence synthesis projects may want to also tap into additional data sources.

### Programming language

Programming languages that are suitable to be used in combination with scholarly APIs include Python, Unix Shell, Matlab, Mathematica, R, and C. The procedure described in this paper is based on Unix Shell (Bash running in a MacOS environment).

### Database management system

The database management system of choice for managing the retrieved bibliographic data was MySQL (locally installed in a MacOS environment). In order for the code presented here to be useful, one is required to have access to a MySQL database, either remotely or as a local installation. The respective installer files can be found at https://dev.mysql.com/. The respective database schemes and tables are provided in the Supporting Information.

## Procedure

**Fig. 1:**
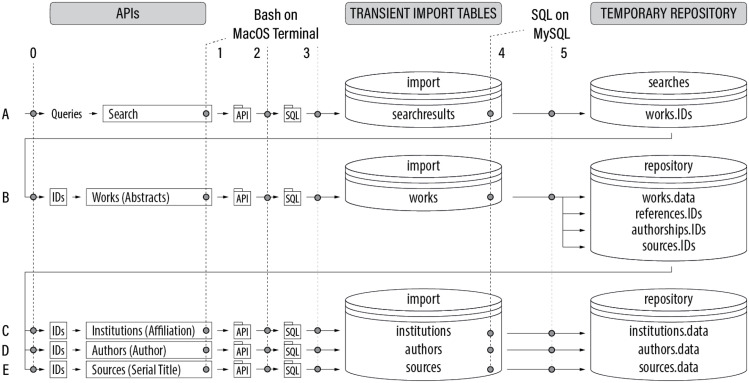
The five stages and six steps in relation to the data sources and database management system (database table names refer to the implementation for OpenAlex; API names refer to OpenAlex with Scopus API names in brackets).

With above choices and preparations in place, the general procedure consists of five stages (A-E) with six steps (0–5) each, as illustrated in Fig. 1. The five stages are outlined in Table 2 and the six steps in Table 3.

**Table 2:** The five stages of the procedure (first line per stage for Scopus, second line per stage for OpenAlex).

Step	Name	API	Description
A	Search Works	Scopus Search API Search Works API	Retrieve search results. *Not implemented.*
B	Retrieve Works	Abstract Retrieval API Works API	Retrieve abstract records. Retrieve works records.
C	Retrieve Institutions	Affiliation Retrieval API Institutions API	Retrieve affiliation records. Retrieve institution records.
D	Retrieve Authors	Author Retrieval API Authors API	Retrieve author records. Retrieve author records.
E	Retrieve Sources	Serial Title Retrieval API Sources API	*Not implemented.* Retrieve source records.

**Table 3:** The five steps from API to temporary repository as basis for further literature analysis and review.

Step	Name	Description
0	Initialisation	Stage A: search queries. Stage B: works (abstract) IDs identified in Stage A. Stage C: institutions (affiliation) IDs extracted in Stage B. Stage D: authors (author) IDs extracted in Stage B. Stage E: source IDs extracted in Stage B.
1	Retrieve Records	Retrieve records using the respective API.
2	Create Insert Statements	Create SQL insert statements to insert the retrieved records into a database.
3	Insert into Database	Actual insertion to the database management system.
4	Process Records	Extract relevant information from the recordsets previously retrieved from Scopus or OpenAlex, respectively, and inserted into the database.
5	Store in Local Repository	Save the information relevant for the analysis locally and temporarily in accordance with the applicable use terms and conditions.

As stated in Table 3, Step 0 in each stage is the initialization step. In Stage A, the input are search queries. In stages B-E, the input are the record IDs retrieved in previous stages. These IDs are saved in text files ‘IDs.txt’, which serve as input for retrieving records in Stages B to E (see Fig. 1).

Steps 1 to 5 are described in broad terms in the remainder of this section. Detailed information per API is provided in the Online Supporting Material.

### Retrieve records

Record retrieval through the API is based on a CURL statement, as shown in Box 1 for Scopus and in Box 2 for OpenAlex. This CURL statement was adjusted in order to be embedded in a Bash batch file intended to loop through all IDs in the input text file ‘IDs.txt’, see Box 3. Note that a small pause is introduced after each record, which is considered good practice. Each record is written to an individual output file in the ‘API’ subfolder.

**Box 1:** CURL statement to retrieve record from API. Example: Elsevier Abstract Retrieval API using eid.

**Box 2:** CURL statement to retrieve record from API. Example: OpenAlex Authors API using authorID.

**Box 3:** Bash batch file to retrieve records from API. Example: Elsevier Abstract Retrieval API using eid.

### Create SQL insert statements

For each output file in the ‘API’ folder, an SQL insert statement is created based on a Bash batch file (see Box 4) and the respective SQL insert statement is saved as file in the ‘SQL’ folder (see Fig. 1).

**Box 4:** Bash batch file to create insert statements. Example: OpenAlex Authors API using authorID.

### Insert records into database management system

Another Bash batch file (see Box 5) then processes files in the 'SQL' folder (see Fig. 1).

**Box 5:** Bash batch for actual database insertion. Example: Generic.

### Extract relevant data elements

**Fig. 2:**
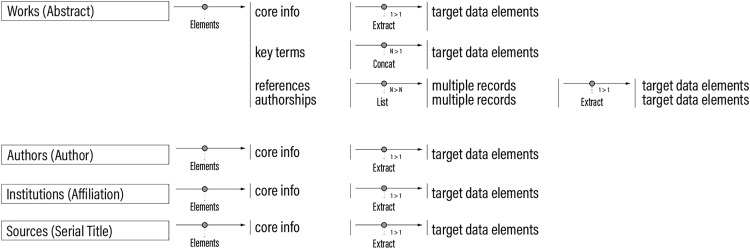
Workflow for extracting target data elements. Focus on data elements to be extracted.

Once records are imported to the database, target data elements are extracted based on the workflow shown in Fig. 2. For author and institution (affiliation) records, all relevant elements can be extracted directly into corresponding columns in the database table. For works (abstract) records, the first step is to split the record based on the JSON or XML structure. Some elements, notably core information (e.g., doi, publication year, title) can be directly extracted to corresponding columns in the database table. Other elements require a loop to process multiple elements. For key terms (e.g., author keywords, index terms), multiple elements are concatenated into an individual corresponding column in the database table. For authors, institutions (affiliations), and references in the bibliography, a row is generated in an auxiliary table for each author, affiliation, or reference, respectively.

Box 6 provides an example of SQL code used to extract core information from abstract records. To streamline processing, individual SQL statements are called from Bash batch files, see Box 7.

**Box 6:** SQL code for extracting data elements. Example: Scopus Abstract Retrieval API.

**Box 7:** Bash batch file to run SQL statements. Example: Scopus Abstract Retrieval API – References.

### Store extracted data elements in local repository for further analysis

**Fig. 3:**
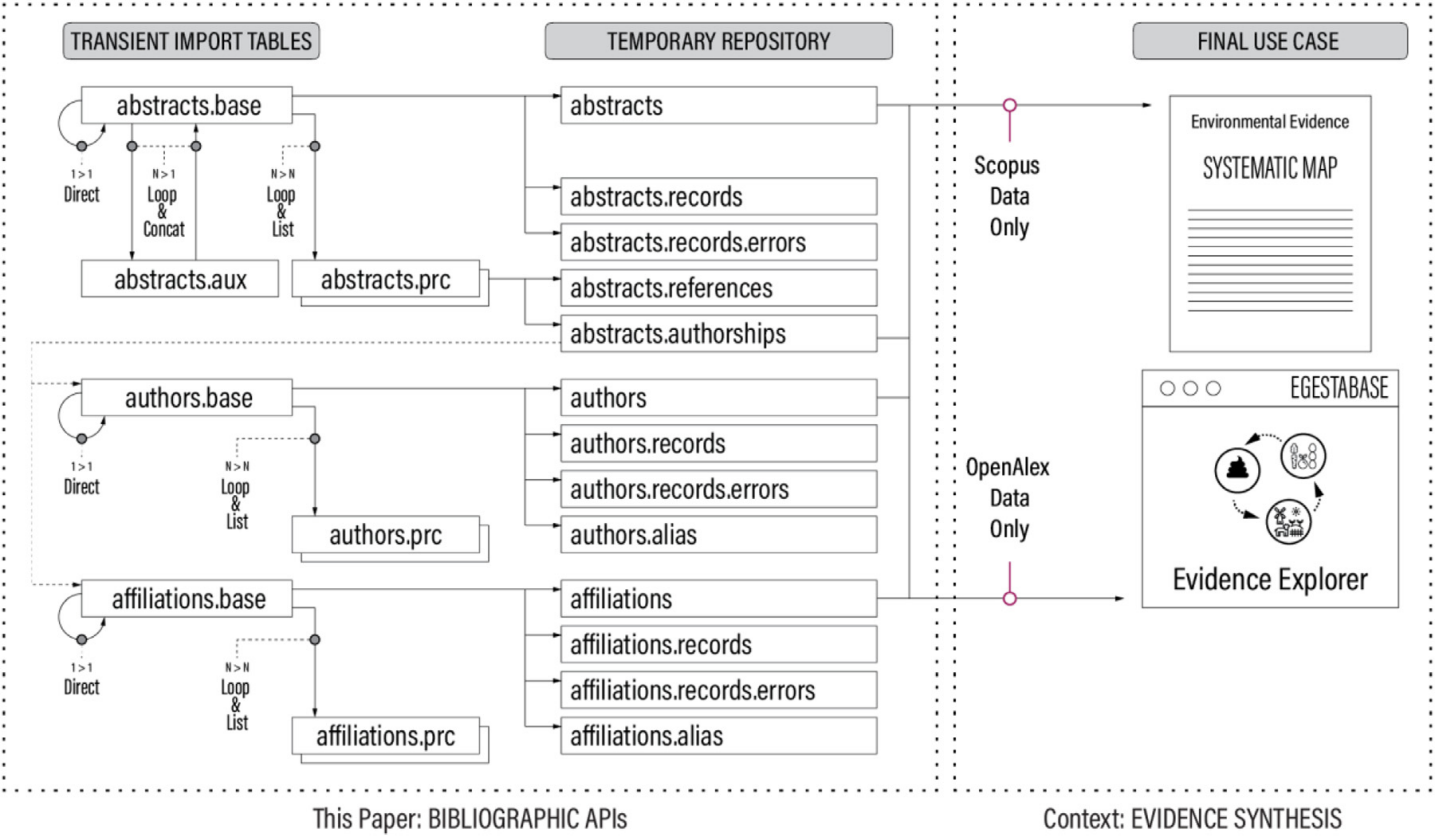
Workflow for extracting and storing target data elements along with retrieved records. Focus on database tables.

For the retrieval APIs, target data elements are stored in a temporary local repository along with the retrieved records (see Fig. 3), which is considered good practice. For the search APIs, only the record IDs of the search hits are stored in the local repository (not shown in Fig. 3).

Box 8 provides an example of SQL code used to store data elements in the local repository. Like in the previous step, to streamline processing, individual SQL statements are also called from Bash batch files.

**Box 8:** SQL code to store extracted data in local repository. Example: Scopus Affiliation Retrieval API.

## Challenges and portability

The procedure and code base presented in this paper was very useful to support the evidence synthesis efforts in the project ‘End-of-Wastewater’. In combination with a bespoke screening and coding tool (that was also developed in the project and was fed with the bibliographic data retrieved through the APIs), it allowed us to increase the speed of screening and coding by about tenfold in comparison with using EPPI-Reviewer. However, developing this code was not trivial. Here, a few of the challenges are described along with considerations regarding the portability of the code base.

### Issues with JSON format

MySQL supports a data type JSON, which would be ideally suited to store the JSON data retrieved through the APIs. The advantage of the JSON data type is that it allows for precise navigation across data elements using their position in the JSON structure. However, for both Scopus and OpenAlex, roughly a third of the records did not pass the JSON validation that is performed automatically when attempting to store a value in a JSON field. This meant that the retrieved records had to be stored as LONGTEXT instead. Without the added benefit of simple and precise navigation throughout the JSON structure, individual data elements had to be extracted with the help of search terms that confine the target data element to the left and right. Finding the right search terms was an iterative process, until it was precise enough not to extract at the wrong place in the JSON structure. For the Scopus Abstract API, this proved to be such a challenge that the solution was to resort to retrieving records in XML rather than JSON format. The XML format features data element delimiters that are easier to target in plain text than JSON data element delimiters.

### OpenAlex still under development

OpenAlex was still under development while the API retrieval code base was written. In the beginning, journals and so forth were referred to as ‘venue’ rather than ‘source’. At some point, ‘venues’ were discontinued and became ‘sources’. This meant that part of the code had to be adjusted to reflect these changes. Also, at some point there were changes to author and institution IDs, which meant that the complete set of records had to be downloaded once more in order to get a fully consistent recordset.

### Performance of the SQL queries

The SQL queries were developed with a sole focus on correctly extracting the target data elements from the retrieved records. Aspects of performance were not considered. The procedure and code work well for up to approximately 10′000 records at a time. Running more than this number of records at a time comes with the risk of hitting MySQL connection timeout limitations. In order to process more than 10′000 records, it is advised to split processing into chunks of no more than 10′000 records at a time.

### Quality of the data extraction

The data extraction procedure has undergone a number of iterations, which meant that a number of small errors and bugs were successively discovered and fixed. In the current form, the procedure should be rather robust in correctly extracting the target data elements. However, it cannot be guaranteed that the data extraction procedure is absolutely fault-proof.

### Portability and adaptation of the procedure and code base

The code base was developed in a MacOS environment with MySQL running locally. In principle, both SQL and Bash code should also run on UNIX or Windows platforms. However, this was not tested. But with this documentation of the procedure and the code base in hand, the seasoned reader should be able to adapt and adjust the procedure and code base for different needs.

## Ethics statements

This work did not involve human subjects, animal experiments, or data collected from social media platforms.

## CRediT authorship contribution statement

**Robin Harder:** Conceptualization, Methodology, Software, Data curation, Writing – original draft, Visualization, Project administration, Funding acquisition.

## Data Availability

No data was used for the research described in the article.

## Appendix Supplementary materials

Image, application 1

Image, application 2

Image, application 3
