# Supplementary material for: Using Scopus and OpenAlex APIs to retrieve bibliographic data for evidence synthesis. A procedure based on Bash and SQL
Source: MethodsX. 2024 Feb 3;12:102601. doi: 10.1016/j.mex.2024.102601 (PMC10867663; doi:10.1016/j.mex.2024.102601)
Supplement: Supplementary file 1 [file mmc1.pdf]

DOI: 10.1016/j.mex.2024.102601

Using Scopus and OpenAlex APIs to retrieve bibliographic data for evidence synthesis. A procedure based on Bash and SQL.

Robin Harder[1]

[1] Environmental Engineering Group, Department of Energy and Technology, Swedish University of Agricultural Sciences (SLU), Uppsala, Sweden

SUPPLEMENTARY MATERIAL 1

Scopus Search API

Table of Content

1 Overall Workflow 2
2 Retrieve Search Result Records 2
3 Load Records into Database Management System 3
4 Extract and Store Target Data 4

N.B.:
The code described here is available from DOI: 10.17632/b4j39ccj8t.1

1 Overall Workflow

The overall workflow for stage A (i.e., literature search, as per Table 2 in the main paper) consists of repeating the six steps (i.e., steps 0 to 5, as per Table 3 in the main paper) for two iterations (as per Figure 1 in this SM). The first iteration (A-1) is initialized by the search strings as specified in the database table 'eow_search_strings' (A-1.0). The second iteration (A-2) is required because result pagination limits the search result record to 25 hits and only applies to search strings yielding more than 25 hits. Each step is facilitated by a suite of batch terminal files, as listed in Figure 1. Details for each step are provided in the remainder of this SM.

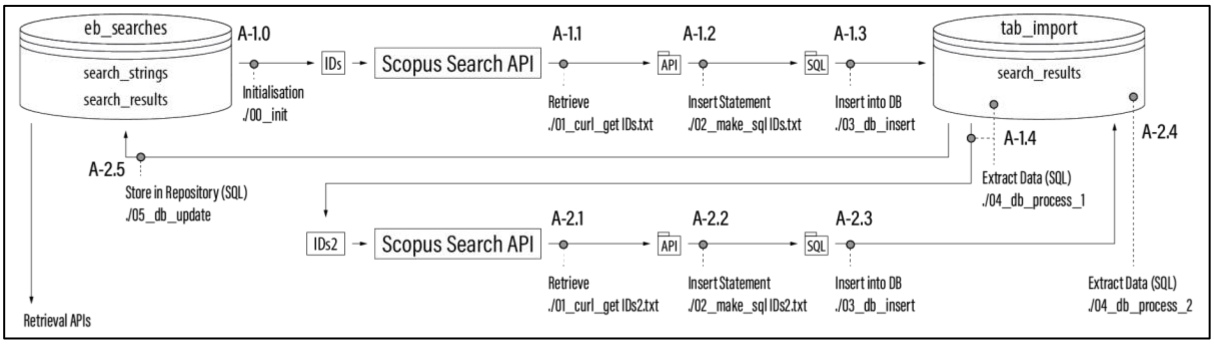

Figure 1: Overall workflow for the literature search (stage A as per Table 2 in the main paper).

2 Retrieve Search Result Records

Step 1 uses MacOS terminal to retrieve individual records using the Scopus Seach API. The general structure of the CURL statements is as follows.

Request URL

```
https://api.elsevier.com/content/search/scopus?query=<query>?apiKey=<apiKey>
```

CURL – without date constraints

```
curl -X GET --header 'Accept: application/xml'
'https://api.elsevier.com/content/search/scopus?query=<query>?apiKey=<apiKey>'
```

CURL – with date constraints

```
curl -X GET --header 'Accept: application/xml'
'https://api.elsevier.com/content/search/scopus?query=<query>+AND+orig-load-
date+aft+YYYYMMDD+AND+NOT+orig-load-date+aft+YYYYMMDD?apiKey=<apiKey>'
```

2.1 General Structure

2.1.1 Bash Batch File

Opening

```
#!/bin/bash
```

Define API Key

```
apikey="<apiKey>"
```

Set Variables

```
x=0
y1=0
y2=0
z1=99
z2=999
```

Core

```
i=0
while IFS= read -r line || [[ -n "$line" ]]
do
        ((i++))
        seq=$(printf "%06d" $i)
        echo "${seq} | EID: ${line}"

        curl -X GET --header 'Accept: application/json'
        'https://api.elsevier.com/content/search/scopus?query='"$line"'&apiKey='"$apikey"
        -o api/$seq.txt

        sleep 0.1
        if [[ $y1 -gt $z1 ]]
        then
        y1=0
        sleep 1
        fi
        if [[ $y2 -gt $z2 ]]
        then
        y2=0
        sleep 10
        fi
done < "$1"
```

2.1.2 Bash Execute Statement

First Iteration

```
./01_curl_get IDs.txt
```

Second Iteration

```
./01_curl_get IDs2.txt
```

Input File Structure – IDs.txt

```
TITLE((urine%20OR%20yellowwater%20OR%20%22yellow%20water%22)%20AND%20(struvite))
TITLE((feces%20OR%20brownwater%20OR%20%22brown%20water%22)%20AND%20(compost))
…
```

Input File Structure – IDs2.txt

```
start=25&TITLE((urine%20OR%20yellowwater%20OR%20%22yellow%20water%22)%20AND%20(struvite))
start=50&TITLE((urine%20OR%20yellowwater%20OR%20%22yellow%20water%22)%20AND%20(struvite))
start=70&TITLE((urine%20OR%20yellowwater%20OR%20%22yellow%20water%22)%20AND%20(struvite))
…
start=25&TITLE((feces%20OR%20brownwater%20OR%20%22brown%20water%22)%20AND%20(compost))
start=50&TITLE((feces%20OR%20brownwater%20OR%20%22brown%20water%22)%20AND%20(compost))
…
…
```

3 Load Records into Database Management System

Step 2 creates an SQL insert statement for each record that was retrieved through the Scopus Search API. Step 3 then executes the actual insertion into the database.

3.1 Create SQL Insert Statements

The general form of the SQL insert statement is as follows.

```
INSERT IGNORE INTO elsevier_scp_api_sr_imp (sequence, query_val, query_result)
VALUES (<sequence>, <query_val>, <query_result>);
```

The base import tables is as follows.

**API**	**Scheme**	**Table**	**Description**
Scopus Search	tab_import_api	elsevier_scp_api_sr_imp	Base import table for search results

The insert variables are as follows.

**Variable**	**Description**	**Examples**
sequence	Sequence number in search strings list	1,2, 3, etc.
query_val	Value queried	see input file structure above
query_result	API response body	…

3.1.1 Bash Batch File

Opening

```
#!/bin/bash
```

Core

```
i=0
while IFS= read -r line || [[ -n "$line" ]]
do
        ((i++))
        seq=$(printf "%06d" $i)
        echo "${seq} | ${line}"
        > sql/sql_$seq.txt
        echo "INSERT IGNORE INTO elsevier_scp_api_sr_imp (sequence, query_val,
        query_result)
        VALUES ('"$seq"', '"$line"', '" >> sql/sql_$seq.txt
        cat api/$seq.txt | sed "s/\'/\\\'/g" >> sql/sql_$seq.txt
        echo "'); " >> sql/sql_$seq.txt
done < "$1"
```

3.1.2 Bash Execute Statement

First Iteration

```
./02_make_sql IDs.txt
```

Second Iteration

```
./02_make_sql IDs2.txt
```

3.2 Import to Database Management System

3.2.1 Bash Batch File

Opening

```
#!/bin/bash
```

Core

```
export MYSQL_PWD=<password>
i=0
for filename in sql/*.txt; do
        ((i++))
        seq=$(printf "%06d" $i)
        xbase=${filename##*/}
        line=${xbase%.*}
        echo "$seq"
        mysql --host=localhost --user=repository tab_import_api < sql/$line.txt
done
```

3.2.2 Bash Execute Statement

First and Second Iteration

```
./03_db_insert
```

4 Extract and Store Target Data

Step 4 extracts target bibliographic data elements for each record in the recordsets previously retrieved from the Scopus Search API and inserted to the database management system. Step 5 then is about storing the extracted target data elements in a temporary local data repository.

4.1 Data Element Targeting

The extraction of target data elements relies on finding specific tags in the JSON structure and requires a number of auxiliary tables, see below.

Specific Tags for Data Element Targeting in Base Import Table

Column	#	Subset	Tag
searchTerms	1	query_result	"opensearch:Query": …
docTotal	1	query_result	"opensearch:totalResults": …
docReturn	1	query_result	***# of*** "eid":"2-s2.0-
pageStart	1	query_result	"opensearch:startIndex":
pageItems	1	query_result	"opensearch:itemsPerPage":"

Auxiliary Tables

Scheme	Table	Description
tab_import_api	elsevier_scp_api_sr_prc_1	Auxiliary table for processing search results
tab_import_api	elsevier_scp_api_sr_prc_2	Auxiliary table for processing search results
tab_import_api	elsevier_scp_api_sr_prc_3	Auxiliary table for processing search results
tab_import_api	elsevier_scp_api_sr_prc_4	Auxiliary table for processing search results
tab_import_api	elsevier_scp_api_sr_prc_5	Auxiliary table for processing search results

Specific Tags for Data Element Targeting in Auxiliary Tables

Column	#	Subset	Tag
eid	1	query_result	"eid":"2-s2.0- … "

4.2 Stored Procedures

Extracting target data elements and storing them in the local repository is facilitated by stored procedures, see below.

Stored Procedures for Extracting Target Data Elements

Scheme	Stored Procedure	Iteration	Description
tab_import_api	elsevier_scp_api_sr_prc_0_01	1	Initialise first iteration with original search strings in CURL format
tab_import_api	elsevier_scp_api_sr_prc_1_00	1	Delete service errors
tab_import_api	elsevier_scp_api_sr_prc_1_01	1	Insert retrieved records into auxiliary table 1
tab_import_api	elsevier_scp_api_sr_prc_1_02	1	Extract target data elements on the level or search hit records
tab_import_api	elsevier_scp_api_sr_prc_1_03	1	Count search hits per search hit record
tab_import_api	elsevier_scp_api_sr_prc_1_04	1	Where search hits >25: Create additional search strings in aux table 2
tab_import_api	elsevier_scp_api_sr_prc_2_01	1	Initialise second iteration with additional search strings in CURL format
tab_import_api	elsevier_scp_api_sr_prc_3_00	2	Insert retrieved additional records into auxiliary table 3
tab_import_api	elsevier_scp_api_sr_prc_3_01	2	Extract target data elements on the level or search hit records
tab_import_api	elsevier_scp_api_sr_prc_4_00	2	Truncate auxiliary table 4
tab_import_api	elsevier_scp_api_sr_prc_4_01	2	Insert records from first iteration
tab_import_api	elsevier_scp_api_sr_prc_4_02	2	Insert records from second iteration
tab_import_api	elsevier_scp_api_sr_prc_5_00	2	Truncate auxiliary table 5
tab_import_api	elsevier_scp_api_sr_prc_5_01	2	Extract search hits

Stored Procedures for Storing Extracted Target Data Elements

Scheme	Stored Procedure	Description
tab_import_api	elsevier_scp_api_sr_upd_01	Store extracted search hits in temporary local repository

Specific Tables for Storing Extracted Target Data Elements

Scheme	Table	Description
eb_searches	eow_results_eidapi	List of search hits per search string

4.3 Bash Files

Calling stored procedures is facilitated by a suite of Bash files, see below.

Folder	Bash File	Description
SCOPUS_SEARCH	04_db_process_1	Run target element extraction and processing – First iteration
SCOPUS_SEARCH	04_db_process_2	Run target element extraction and processing – Second iteration
SCOPUS_SEARCH	05_db_update	Update local temporary repository
