# Supplementary material for: Using Scopus and OpenAlex APIs to retrieve bibliographic data for evidence synthesis. A procedure based on Bash and SQL
Source: MethodsX. 2024 Feb 3;12:102601. doi: 10.1016/j.mex.2024.102601 (PMC10867663; doi:10.1016/j.mex.2024.102601)
Supplement: Supplementary file 2 [file mmc2.pdf]

DOI: 10.1016/j.mex.2024.102601

Using Scopus and OpenAlex APIs to retrieve bibliographic data for evidence synthesis. A procedure based on Bash and SQL.

Robin Harder[1]

[1] Environmental Engineering Group, Department of Energy and Technology, Swedish University of Agricultural Sciences (SLU), Uppsala, Sweden

SUPPLEMENTARY MATERIAL 2

Scopus Retrieval APIs

Table of Content

1 Overall Workflow 2
2 Retrieve Data from Scopus APIs 2
3 Load Records into Database Management System 4
4 Extract and Store Target Data 7

N.B.:
The code described here is available from DOI: 10.17632/b4j39ccj8t.1

1 Overall Workflow

The overall workflow consists of repeating the six steps (i.e., 0 to 5, as per Table 3 in the main paper) across stages B to D (as per Table 2 in the main paper). Stage B (retrieve abstracts) is initialized by the search results from stage A. Stages C (retrieve affiliations) and D (retrieve authors) are initialized by taking the affiliation and author IDs, respectively, as extracted from the abstracts retrieved in Stage B. Each step is facilitated by a suite of batch terminal files, as listed in the Figure below. Details for each step are provided in the remainder of this SM.

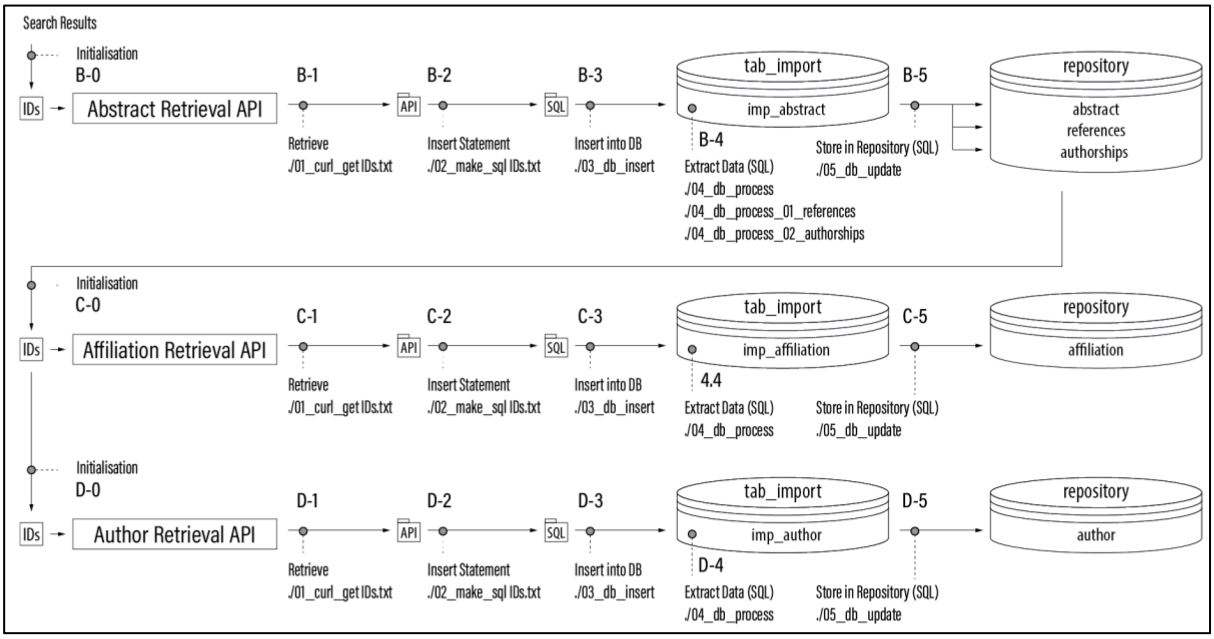

2 Retrieve Data from Scopus APIs

Step 1 uses MacOS terminal to retrieve individual records using Scopus APIs. The general structure of the CURL statements is as follows.

Abstract Retrieval API

Request URL

```
https://api.elsevier.com/content/abstract/scopus_id/85060852025?apiKey=<apiKey>
```

CURL with eid

```
curl -X GET --header 'Accept: application/xml'
'https://api.elsevier.com/content/abstract/eid/2-s2.0-85060852025?apiKey=<apiKey>'
```

CURL with scopusID

```
curl -X GET --header 'Accept: application/xml'
'https://api.elsevier.com/content/abstract/scopus_id/85060852025?apiKey=<apiKey>'
```

CURL with pubmedID

```
curl -X GET --header 'Accept: application/xml'
'https://api.elsevier.com/content/abstract/pubmed_id/32203816?apiKey=<apiKey>'
```

CURL with pii

```
curl -X GET --header 'Accept: application/xml'
'https://api.elsevier.com/content/abstract/pii/S0921344921004511?apiKey=<apiKey>'
```

CURL with doi

```
curl -X GET --header 'Accept: application/xml'
'https://api.elsevier.com/content/abstract/doi/10.1080/10643389.2018.1558889?apiKey=<apiKey>'
```

Author Retrieval API

Request URL

```
https://api.elsevier.com/content/author/author_id/55413737300?apiKey=<apiKey>
```

CURL with authorID

```
curl -X GET --header 'Accept: application/json'
'https://api.elsevier.com/content/author/author_id/55413737300?apiKey=<apiKey>'
```

Affiliation Retrieval API

Request URL

```
https://api.elsevier.com/content/affiliation/affiliation_id/60000990?apiKey=<apiKey>
```

CURL with affiliationID

```
curl -X GET --header 'Accept: application/json'
'https://api.elsevier.com/content/affiliation/affiliation_id/60000990?apiKey=<apiKey>'
```

2.1 General Structure

2.1.1 Bash Batch File

Opening

```
#!/bin/bash
```

Define API Key

```
apikey="<apiKey>"
```

Define and Select ID Types

```
<Only for Abstract Retrieval API – see below>
```

Set Variables

```
x=0

y1=0
y2=0
z1=99
z2=999
```

Core

```
i=0
while IFS= read -r line || [[ -n "$line" ]]
do
        ((i++))
        seq=$(printf "%06d" $i)

        <Specific Code Elements per API – see below>

        sleep 0.2
        if [[ $y1 -gt $z1 ]]
        then
        y1=0
        sleep 2
        fi
        if [[ $y2 -gt $z2 ]]
        then
        y2=0
        sleep 10
        fi
done < "$1"
```

2.1.2 Bash Execute Statement

```
./01_curl_get IDs.txt
```

2.2 Specific Code Elements per API

2.2.1 *Abstract Retrieval API*

Define and Select ID Types

```
PS2='Please choose type of identifier: '
options=("EID" "PII" "DOI" "PMID")
select opt in "${options[@]}"
do
case $opt in
"EID")
identifier="EID"
        echo "$identifier"
        break
;;
"DOI")
identifier="DOI"
        echo "$identifier"
        break
;;
<dito for PII and PMID>
*) echo "invalid option $REPLY";;
esac
done
```

Core

```
if [[ $identifier == "EID" ]]
then
echo "${seq} | EID: ${line}"
curl -X GET --header 'Accept: application/xml'
'https://api.elsevier.com/content/abstract/eid/2-s2.0-'"$line"'?apiKey='"$apikey" -o
api/$seq.txt
elif [[ $identifier == "DOI" ]]
then
echo "${seq} | DOI: ${line}"
curl -X GET --header 'Accept: application/xml'
'https://api.elsevier.com/content/abstract/doi/'"$line"'?apiKey='"$apikey" -o api/$seq.txt
<dito for PII and PMID>
else
echo "ERROR"
fi
```

2.2.2 *Author Retrieval API*

Core

```
echo "${seq} | AUID: ${line}"
curl -X GET --header 'Accept: application/json'
'https://api.elsevier.com/content/author/author_id/'"$line"'?apiKey='"$apikey" -o api/$seq.txt
```

2.2.3 *Affiliation Retrieval API*

Core

```
echo "${seq} | AFID: ${line}"
curl -X GET --header 'Accept: application/json'
'https://api.elsevier.com/content/affiliation/affiliation_id/'"$line"'?apiKey='"$apikey" -o
api/$seq.txt
```

3 Load Records into Database Management System

Step 2 creates an SQL insert statement for each record that was retrieved through the respective Scopus API. Step 3 then executes the actual insertion into the database.

The general form of the insert statement is as follows.

```
INSERT IGNORE INTO <base_import_table> (sequence, query_key, query_val, query_result)
VALUES (<sequence>, <query_key>, <query_val>, <query_result>);
```

The base import tables per API are as follows.

API	Scheme	Table	Description
Author Retrieval	tab_import_api	elsevier_scp_api_au_imp	Base import table for authors
Affiliation Retrieval	tab_import_api	elsevier_scp_api_af_imp	Base import table for affiliations
Abstract Retrieval	tab_import_api	elsevier_scp_api_ab_imp	Base import table for abstracts

The variables are as follows.

Variable	Description	Examples
sequence	Sequence number in a list of IDs to be queried.	1,2, 3, etc.
query_key	Type of ID queried.	eid, doi, author_id, affiliation_id, etc.
query_val	Value of the id queried	85060852025, S0921344921004511, etc.
query_result	API response body	…

3.1 Create SQL Insert Statements – General Structure

3.1.1 Bash Batch File

Opening

```
#!/bin/bash
```

Define and Select ID Types

```
<Only for Abstract Retrieval API - see below>
```

Core

```
i=0
while IFS= read -r line || [[ -n "$line" ]]
do
        ((i++))
        seq=$(printf "%06d" $i)
        <Specific per API - see below>
done < "$1"
```

3.1.2 Bash Execute Statement

In Terminal

```
./02_make_sql IDs.txt
```

IDs.txt Input File Structure

```
85060852025
…
```

3.2 Create SQL Insert Statements – Specific Elements per API

3.2.1 Abstract Retrieval API

Define and Select ID Types

```
PS2='Please choose type of identifier: '
options=("EID" "PII" "DOI" "PMID")
select opt in "${options[@]}"
do
case $opt in
"EID")
identifier="EID"
        echo "$identifier"
        break
;;
"DOI")
identifier="DOI"
        echo "$identifier"
        break
;;
<dito for PII and PMID>
*) echo "invalid option $REPLY";;
esac
done
```

Core

```
if [[ $identifier == "EID" ]]
then
echo "${seq} | EID: ${line}"
query_key="EID"
elif [[ $identifier == "DOI" ]]
then
echo "${seq} | DOI: ${line}"
query_key="DOI"
<dito for PII and PMID>
else
echo "ERROR"
fi
> sql/sql_$seq.txt
echo "INSERT IGNORE INTO elsevier_scp_api_ab_imp (sequence, query_key, query_val,
query_result) VALUES ('"$seq"', '"$query_key"', '"$line"', '" >> sql/sql_$seq.txt
cat api/$seq.txt | sed "s/\'/\\\'/g" >> sql/sql_$seq.txt
echo "'); " >> sql/sql_$seq.txt
```

3.2.2 Author Retrieval API

Core

```
echo "${seq} | AUID: ${line}"
query_key="AUID"
> sql/sql_$seq.txt
echo "INSERT IGNORE INTO elsevier_scp_api_au_imp (sequence, query_key, query_val,
query_result) VALUES ('"$seq"', '"$query_key"', '"$line"', '" >> sql/sql_$seq.txt
cat api/$seq.txt | sed "s/\'/\\\'/g" >> sql/sql_$seq.txt
echo "'); " >> sql/sql_$seq.txt
```

3.2.3 Affiliation Retrieval API

Core

```
echo "${seq} | AFID: ${line}"
query_key="AFID"
> sql/sql_$seq.txt
echo "INSERT IGNORE INTO elsevier_scp_api_af_imp (sequence, query_key, query_val,
query_result) VALUES ('"$seq"', '"$query_key"', '"$line"', '" >> sql/sql_$seq.txt
cat api/$seq.txt | sed "s/\'/\\\'/g" >> sql/sql_$seq.txt
echo "'); " >> sql/sql_$seq.txt
```

3.3 Import to Database Management System

3.3.1 Bash Batch File

Opening

```
#!/bin/bash
```

Core

```
export MYSQL_PWD=<password>
i=0
for filename in sql/*.txt; do
        ((i++))
        seq=$(printf "%06d" $i)
        xbase=${filename##*/}
        line=${xbase%.*}
        echo "$seq"
        mysql --host=localhost --user=repository tab_import_api < sql/$line.txt
done
```

3.3.2 Bash Execute Statement

In Terminal

```
./03_db_insert
```

4 Extract and Store Target Data

Step 4 extracts relevant bibliographic data elements from the recordsets previously retrieved from the Scopus APIs and inserted to the database. Step 5 then is about storing the extracted data elements in a temporary local data repository. Data extraction takes place as detailed in the Figure below. Note that loops are required for target data elements with multiple elements. Multiple author keywords, index terms, and subject areas are concatenated into one column in the respective auxiliary database table. Multiple references in the bibliography, as well as, multiple affiliations and authors are stored as separate records in auxiliary database tables.

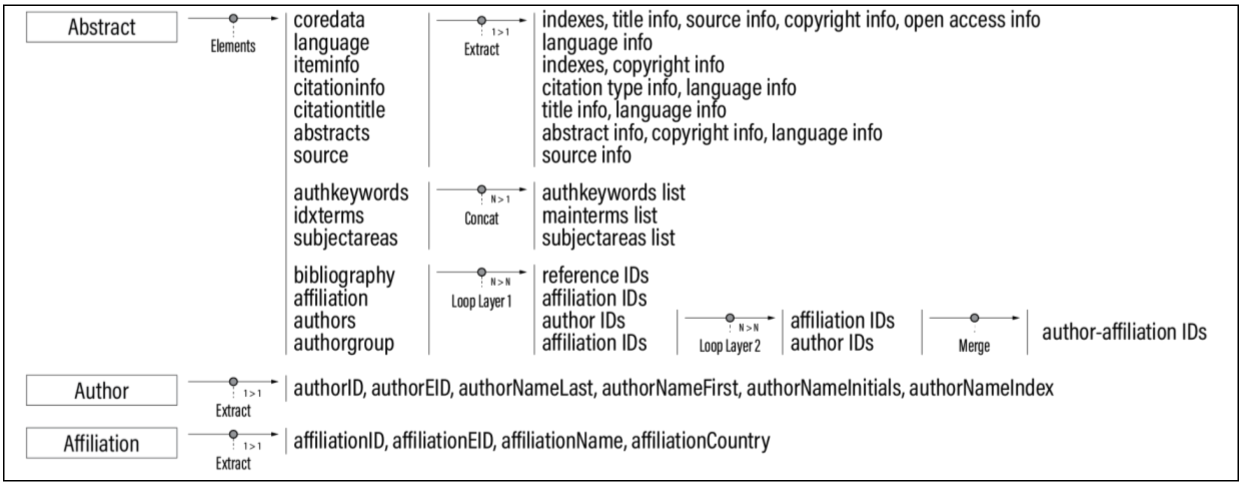

4.1 Data Extraction – Auxiliary Tables

In addition to the base import tables, the extraction of target bibliographic data elements requires a number of auxiliary tables, which are listed below per API.

4.1.1 Abstract Retrieval API

Scheme	Table	Description
tab_import_api	elsevier_scp_api_ab_imp_aux	Generic auxiliary table for processing various elements
tab_import_api	elsevier_scp_api_ab_prc_affiliation	Auxiliary table for processing affiliations
tab_import_api	elsevier_scp_api_ab_prc_author_1	Auxiliary table for processing authors
tab_import_api	elsevier_scp_api_ab_prc_author_2	Auxiliary table for processing authors
tab_import_api	elsevier_scp_api_ab_prc_authorgroup_1	Auxiliary table for processing author groups
tab_import_api	elsevier_scp_api_ab_prc_authorgroup_2	Auxiliary table for processing author groups
tab_import_api	elsevier_scp_api_ab_prc_authorship	Auxiliary table for processing authorships
tab_import_api	elsevier_scp_api_ab_prc_references	Auxiliary table for processing references

4.1.2 Author Retrieval API

Scheme	Table	Description
tab_import_api	elsevier_scp_api_au_imp_alias	Auxiliary table for processing authors (aliases)
tab_import_api	elsevier_scp_api_au_prc_alias_1	Auxiliary table for processing authors (aliases)
tab_import_api	elsevier_scp_api_au_prc_alias_2	Auxiliary table for processing authors (aliases)

4.1.3 Affiliation Retrieval API

Scheme	Table	Description
tab_import_api	---	No specific auxiliary table for processing affiliations

4.2 Data Extraction – Search Tags for Data Element Targeting

The extraction of target bibliographic data elements is done based on finding specific tags in the record. For the abstract retrieval API, this relied on XML tags. For the author and affiliation retrieval APIs, this relied on the JSON structure.

4.2.1 Abstract Retrieval API

Base Import Table (XML)

Column	#	Subset	Tag
sgr	1	xml_coredata	<eid> ... </eid>
eid	1	xml_coredata	<eid> ... </eid>
doi	1	xml_coredata	<prism:doi> ... </prism:doi>
pii	1	xml_coredata	<pii> ... </pii>
pmid	1	xml_coredata	<pubmed-id> ... </pubmed-id>
core_srctype	1	xml_coredata	<srctype> ... </srctype>
core_subtype	1	xml_coredata	<subtype> ... </subtype>
core_subtypeDescription	1	xml_coredata	<subtypeDescription> ... </subtypeDescription>
core_citedbycount	1	xml_coredata	<citedby-count> ... </citedby-count>
core_openaccess	1	xml_coredata	<openaccess> ... </openaccess>
core_openaccessFlag	1	xml_coredata	<openaccessFlag> ... </openaccessFlag>
core_dc_identifier	1	xml_coredata	<dc:identifier> ... </dc:identifier>
core_dc_title	1	xml_coredata	<dc:title> ... </dc:title>
core_dc_publisher	1	xml_coredata	<dc:publisher> ... </dc:publisher>
core_dc_abstract_copy	1	xml_coredata	<publishercopyright> ... </publishercopyright>
core_dc_abstract_para	1	xml_coredata	<ce:para> ... </ce:para>
core_prism_aggregationtype	1	xml_coredata	<prism:aggregationType> ... </prism:aggregationType>
core_prism_publicationname	1	xml_coredata	<prism:publicationName> ... </prism:publicationName>
core_prism_sourceid	1	xml_coredata	<source-id> ... </source-id>
core_prism_issn	1	xml_coredata	<prism:issn> ... </prism:issn>
core_prism_volume	1	xml_coredata	<prism:volume> ... </prism:volume>
core_prism_issueIdentifier	1	xml_coredata	<prism:issueIdentifier> ... </prism:issueIdentifier>
core_prism_pageStarting	1	xml_coredata	<prism:startingPage> ... </prism:startingPage>
core_prism_pageEnding	1	xml_coredata	<prism:endingPage> ... </prism:endingPage>
core_prism_pageRange	1	xml_coredata	<prism:pageRange> ... </prism:pageRange>
core_prism_coverDate	1	xml_coredata	<prism:coverDate> ... </prism:coverDate>
language_xml	1	xml_language	<language xml:lang = " ... ">
authorkeywords	N>1	xml_authkeywords	<author-keywords> ... </author-keywords>
idxterms	N>1	xml_idxterms	<idxterms> ... </idxterms>
subjectareas	N>1	xml_subjectareas	<subject-areas> ... </subject-areas>
iteminfo_copyright_type	1	xml_iteminfo	<copyright type = " … ">
iteminfo_copyright_text	1	xml_iteminfo	<copyright> ... </copyright>
iteminfo_ce_doi	1	xml_iteminfo	<ce:doi> ... </ce:doi>
iteminfo_ce_pii	1	xml_iteminfo	<ce:pii> ... </ce:pii>
iteminfo_scp	1	xml_iteminfo	<itemid type="SCP"> ... </itemid>
iteminfo_sgr	1	xml_iteminfo	<itemid type="SGR"> ... </itemid>
citationinfo_type	1	xml_citationinfo	<citation-type>
citationinfo_citation_language	1	xml_citationinfo	<citation-language language = " ... ">
citationinfo_citation_language_xml	1	xml_citationinfo	<citation-language xml:lang = " ... ">
citationinfo_abstract_language	1	xml_citationinfo	<abstract-language language = " ... ">
citationinfo_abstract_language_xml	1	xml_citationinfo	<abstract-language xml:lang = " ... ">
citationtitle_language	1	xml_citationtitle	<titletext language = " ... ">
citationtitle_language_xml	1	xml_citationtitle	<titletext xml:lang = " ... ">
citationtitle_title	1	xml_citationtitle	<titletext> ... </titletext>
abstract_language_xml	1	xml_abstracts	<abstract xml:lang = " ... ">
abstract_copy	1	xml_abstracts	<publishercopyright> ... </publishercopyright>
abstract_para	1	xml_abstracts	<ce:para> ... </ce:para>
source_srcid	1	xml_source	<source srcid = "…">
source_type	1	xml_source	<source type = "…">
source_title	1	xml_source	<sourcetitle> ... </sourcetitle>
source_title_abbrev	1	xml_source	<sourcetitle-abbrev> ... </sourcetitle-abbrev>
source_issn_print	1	xml_source	<issn type="print"> ... </issn>
source_issn_electronic	1	xml_source	<issn type="electronic"> ... </issn>
source_publicationyear	1	xml_source	<publicationyear first = " ... ">
source_publisher	1	xml_source	<publishername> ... </publishername>
source_volume	1	xml_source	<voliss volume = " ... ">
source_issue	1	xml_source	<voliss issue = " ... ">
source_pageFirst	1	xml_source	<pagerange first = " ... ">
source_pageLast	1	xml_source	<pagerange last = " ... ">
author_first	1	xml_authors	<author seq ="1" auid = " … ">
author_second	1	xml_authors	<author seq ="2" auid = " … ">
author_third	1	xml_authors	<author seq ="3" auid = " … ">
author_citation	1	xml_authors	“Author 1” OR “Author 1 and Author 2” OR “Author 1 et al”
bib_refcount	1	xml_bibliography	<bibliography refcount = " ... ">
count_references	1	xml_bibliography	# </reference>
count_affiliation	1	xml_affiliation	# </affiliation>
count_authors	1	xml_authors	# </author>
count_authorgroup	1	xml_authorgroup	# </affiliation>

Auxiliary Table for References (XML)

Column	#	Subset	Tag
eid		xml_coredata	
sgr		xml_coredata	
seq	1	*Auto Increment*	
ref_id	1	xml_bibliography	<reference id = " ... ">
ref_sgr	1	xml_bibliography	<itemid idtype="SGR"> … </itemid>

Auxiliary Table for Affiliations (XML)

Column	#	Subset	Tag
eid		xml_coredata	
sgr		xml_coredata	
seq	1	*Auto Increment*	
af_seq	1	*Auto Increment*	
af_afid	1	xml_affiliation	<affiliation> ... </affiliation>
af_name	1	xml_affiliation	<affilname id = " ... ">
af_city	1	xml_affiliation	<affiliation-city> ... </affiliation-city>
af_country	1	xml_affiliation	<affiliation-country> ... </affiliation-country>

Auxiliary Table for Authors (XML)

Column	#	Subset	Tag
eid		xml_coredata	
sgr		xml_coredata	
seq_au	1	*Auto Increment*	
seq_af	1	*Auto Increment*	
au_seq	1	xml_authors	<author seq = " ... ">
au_auid	1	xml_authors	<author auid = " ... ">
af_afid	1	xml_authors	<affiliation id = " ... ">

Auxiliary Table for Authorgroups (XML)

Column	#	Subset	Tag
eid		xml_coredata	
sgr		xml_coredata	
seq	1	*Auto Increment*	
au_seq	1	xml_authorgroup	<author seq = " ... ">
au_auid	1	xml_authorgroup	<author id = " ... ">
au_type	1	xml_authorgroup	<author type = " ... ">
af_seq	1	xml_authorgroup	*1*
af_afid	1	xml_authorgroup	<affiliation afid = " ... ">
af_dptid	1	xml_authorgroup	<affiliation dptid = " ... ">
af_country	1	xml_authorgroup	<affiliation country = " ... ">

4.2.2 Author Retrieval API

Base Import Table (JSON)

Column	#	Subset	Tag
au_auid	1	query_result	"AUTHOR_ID: … "
au_eid	1	query_result	"eid": "…"
au_name_last	1	query_result	"surname": "…"
au_name_first	1	query_result	"given-name": "…"
au_name_initials	1	query_result	"initials": "…"
au_name_index	1	query_result	"indexed-name": "…"

4.2.3 Affiliation Retrieval API

Base Import Table (JSON)

Column	#	Subset	Tag
af_afid	1	query_result	"AFFILIATION_ID: … "
af_eid	1	query_result	"eid": "…"
af_name	1	query_result	"affiliation-name": "…"
af_country	1	query_result	"@country": "…"

4.3 Data Extraction – Stored Procedures

Extracting target data elements and storing them in the local repository is facilitated by stored procedures, see below.

4.3.1 Abstract Retrieval API

Scheme	Stored Procedure	Description
tab_import_api	elsevier_scp_api_ab_prc_00_truncate	Empty auxiliary tables
tab_import_api	elsevier_scp_api_ab_prc_01_xml_01_spaces	Remove spaces from XML
tab_import_api	elsevier_scp_api_ab_prc_01_xml_02_split	Split XML into subsets
tab_import_api	elsevier_scp_api_ab_prc_02_data_00_index	Extract coredata elements: indexes (eid, doi, etc)
tab_import_api	elsevier_scp_api_ab_prc_02_data_01_coredata	Extract coredata elemetns: other
tab_import_api	elsevier_scp_api_ab_prc_02_data_02_language	Extract language elements
tab_import_api	elsevier_scp_api_ab_prc_02_data_03_iteminfo	Extract iteminfo elements
tab_import_api	elsevier_scp_api_ab_prc_02_data_04_citation	Extract citationinfo and citationtitle elements
tab_import_api	elsevier_scp_api_ab_prc_02_data_05_abstract	Extract abstract elements
tab_import_api	elsevier_scp_api_ab_prc_02_data_06_source	Extract source elements
tab_import_api	elsevier_scp_api_ab_prc_02_data_07_bibliography	Extract bibliography elements (but not references)
tab_import_api	elsevier_scp_api_ab_prc_02_data_08_authors_01	Extract 1st, 2nd and 3rd authors
tab_import_api	elsevier_scp_api_ab_prc_02_data_08_authors_02	Create author citation (AU1 OR AU1 and AU 2 or AU1 et al.)
tab_import_api	elsevier_scp_api_ab_prc_02_data_09_authkeywords_01	Extract author keywords [Loop]
tab_import_api	elsevier_scp_api_ab_prc_02_data_09_authkeywords_02	Concatenate author keywords
tab_import_api	elsevier_scp_api_ab_prc_02_data_09_idxterms_01	Extract index terms [Loop]
tab_import_api	elsevier_scp_api_ab_prc_02_data_09_idxterms_02	Concatenate index terms
tab_import_api	elsevier_scp_api_ab_prc_02_data_09_subjectareas_01	Extract subject areas [Loop]
tab_import_api	elsevier_scp_api_ab_prc_02_data_09_subjectareas_02	Concatenate subject areas
tab_import_api	elsevier_scp_api_ab_prc_02_data_10_set_status_01	Set status per subset (1 = extracted, 0 = not available)
tab_import_api	elsevier_scp_api_ab_prc_02_data_10_set_status_02	Set overal status (sum of subset status)
tab_import_api	elsevier_scp_api_ab_prc_03_references_01	Count references
tab_import_api	elsevier_scp_api_ab_prc_03_references_02	Extract references to auxiliary table [Loop Layer 1]
tab_import_api	elsevier_scp_api_ab_prc_03_references_03	Extract referring and referred scopusID
tab_import_api	elsevier_scp_api_ab_prc_04_affiliation_01	Count affiliations
tab_import_api	elsevier_scp_api_ab_prc_04_affiliation_02	Extract affiliations to auxiliary table [Loop Layer 1]
tab_import_api	elsevier_scp_api_ab_prc_04_affiliation_03	Extract affiliation elements
tab_import_api	elsevier_scp_api_ab_prc_04_author_01	Count authors
tab_import_api	elsevier_scp_api_ab_prc_04_author_02	Extract authors to auxiliary table [Loop Layer 1]
tab_import_api	elsevier_scp_api_ab_prc_04_author_03	Count authors and affiliations per author instance
tab_import_api	elsevier_scp_api_ab_prc_04_author_04	Extract author affiliations to auxiliary table [Loop Layer 2]
tab_import_api	elsevier_scp_api_ab_prc_04_author_05	Extract author and affiliation elements
tab_import_api	elsevier_scp_api_ab_prc_04_authorgroup_01	Count author-groups
tab_import_api	elsevier_scp_api_ab_prc_04_authorgroup_02	Extract author-groups to auxiliary table [Loop Layer 1]
tab_import_api	elsevier_scp_api_ab_prc_04_authorgroup_03	Count authors and affiliations per author-group instance
tab_import_api	elsevier_scp_api_ab_prc_04_authorgroup_04	Extract author-group authors to auxiliary table [Loop Layer 2]
tab_import_api	elsevier_scp_api_ab_prc_04_authorgroup_05	Extract author and affiliation elements
tab_import_api	elsevier_scp_api_ab_prc_05_authorship_01	Empty authorship auxiliary tables
tab_import_api	elsevier_scp_api_ab_prc_05_authorship_02	Get author-affiliation instances from ‘author’ elements
tab_import_api	elsevier_scp_api_ab_prc_05_authorship_03	Get author-affiliation instances from ‘author-group’ elements

4.3.2 Author Retrieval API

Scheme	Stored Procedure	Description
tab_import_api	elsevier_scp_api_au_prc_00_truncate	Empty auxiliary tables
tab_import_api	elsevier_scp_api_au_prc_01_extract	Extract elements
tab_import_api	elsevier_scp_api_au_prc_02_alias_00	Identify alias records in base import table
tab_import_api	elsevier_scp_api_au_prc_02_alias_01	Extract alias records
tab_import_api	elsevier_scp_api_au_prc_02_alias_02	Extract au_auid and au_auid for alias
tab_import_api	elsevier_scp_api_au_prc_02_alias_03	Write to auxiliary table and loop to next alias

4.3.3 Affiliation Retrieval API

Scheme	Stored Procedure	Description
tab_import_api	elsevier_scp_api_af_prc_00_truncate	Empty auxiliary tables
tab_import_api	elsevier_scp_api_af_prc_01_extract	Extract elements

4.4 Data Storage – Local Repository Tables

For storage of target bibliographic data elements in a local temporary repository, a number of tables were used, see below.

4.4.1 Abstract Retrieval API

Scheme	Table	Description
repository_api_scopus	elsevier_scp_api_abstract	Target data for author records
repository_api_scopus	elsevier_scp_api_abstract_raw	Target data for author records in raw format
repository_api_scopus	elsevier_scp_api_abstract_records	Abstract records
repository_api_scopus	elsevier_scp_api_abstract_records_errors	Errors for abstract records

repository_api_scopus	elsevier_scp_api_authorship	Extracted authorships
repository_api_scopus	elsevier_scp_api_authorship_affiliation	Extracted affiliations
repository_api_scopus	elsevier_scp_api_authorship_author	Extracted authors
repository_api_scopus	elsevier_scp_api_authorship_authorgroup	Extracted authorgroups
repository_api_scopus	elsevier_scp_api_references	Extracted references

4.4.2 Author Retrieval API

Scheme	Table	Description
repository_api_scopus	elsevier_scp_api_author	Author records and target data for author records
repository_api_scopus	elsevier_scp_api_author_error	Errors for author records
repository_api_scopus	elsevier_scp_api_author_alias	Author aliases

4.4.3 Affiliation Retrieval API

Scheme	Table	Description
repository_api_scopus	elsevier_scp_api_affiliation	Affiliation records and target data for affiliation records
repository_api_scopus	elsevier_scp_api_affiliation_error	Errors for affiliation records

4.5 Data Storage – Stored Procedures

Storing target bibliographic data elements in a local temporary repository is facilitated by stored procedures, see below. The actual code base is provided in Appendix A and OSM2.

4.5.1 Abstract Retrieval API

Scheme	Stored Procedure	Description
tab_import_api	elsevier_scp_api_ab_upd_01_bib_data_records	Store data for successfully retrieved records
tab_import_api	elsevier_scp_api_ab_upd_02_bib_data_records_errors	Store error messages
tab_import_api	elsevier_scp_api_ab_upd_03_bib_data_raw	Store extracted data in raw format
tab_import_api	elsevier_scp_api_ab_upd_04_bib_refs	Store extracted references
tab_import_api	elsevier_scp_api_ab_upd_05_bib_refs_status	Store reference extraction status
tab_import_api	elsevier_scp_api_ab_upd_06_bib_data	Store extracted data in adjusted format
tab_import_api	elsevier_scp_api_ab_upd_07_authorship	Store extracted authorships
tab_import_api	elsevier_scp_api_ab_upd_07_authorship_affiliation	Store extracted affiliations
tab_import_api	elsevier_scp_api_ab_upd_07_authorship_author	Store extracted authors
tab_import_api	elsevier_scp_api_ab_upd_07_authorship_authorgroup	Store extracted authorgroups

4.5.2 Author Retrieval API

Scheme	Stored Procedure	Description
tab_import_api	elsevier_scp_api_au_upd_01_insert	Store data for retrieved records and extracted bibliographic data
tab_import_api	elsevier_scp_api_au_upd_01_insert_error	Store error messages
tab_import_api	elsevier_scp_api_au_upd_02_alias	Store author aliases

4.5.3 Affiliation Retrieval API

Scheme	Stored Procedure	Description
tab_import_api	elsevier_scp_api_af_upd_01_insert	Store data for retrieved records and extracted bibliographic data
tab_import_api	elsevier_scp_api_af_upd_01_insert_error	Store error messages

4.6 Bash Files per API

To facilitate smooth and automated calling of the stored procedures, a number of bash batch files were used, see below.

4.6.1 Abstract Retrieval API

Folder	Bash File	Description
SCOPUS_ABSTRACT	04_db_process	Extract core elements
SCOPUS_ABSTRACT	04_db_process_01_references	Extract references
SCOPUS_ABSTRACT	04_db_process_02_authorships	Extract authors, affiliations, and authorgroups
SCOPUS_ABSTRACT	05_db_update	Update local temporary repository

4.6.2 Author Retrieval API

Folder	Bash File	Description
SCOPUS_AUTHOR	04_db_process	Extract core elements
SCOPUS_AUTHOR	05_db_update	Update local temporary repository

4.6.3 Affiliation Retrieval API

Folder	Bash File	Description
SCOPUS_AFFILIATION	04_db_process	Extract core elements
SCOPUS_AFFILIATION	05_db_update	Update local temporary repository
