# Supplementary material for: Using Scopus and OpenAlex APIs to retrieve bibliographic data for evidence synthesis. A procedure based on Bash and SQL
Source: MethodsX. 2024 Feb 3;12:102601. doi: 10.1016/j.mex.2024.102601 (PMC10867663; doi:10.1016/j.mex.2024.102601)
Supplement: Supplementary file 3 [file mmc3.pdf]

DOI: 10.1016/j.mex.2024.102601

Using Scopus and OpenAlex APIs to retrieve bibliographic data for evidence synthesis. A procedure based on Bash and SQL.

Robin Harder[1]

[1] Environmental Engineering Group, Department of Energy and Technology, Swedish University of Agricultural Sciences (SLU), Uppsala, Sweden

SUPPLEMENTARY MATERIAL 3

OpenAlex Retrieval APIs

Table of Content

1 Overall Workflow 2
2 Retrieve Data from OpenAlex APIs 2
3 Load Records into Database Management System 5
4 Extract and Store Target Data 8

N.B.:
The code described here is available from DOI: 10.17632/b4j39ccj8t.1

1 Overall Workflow

The overall workflow consists of repeating the six steps (i.e., 0 to 5, as per Table 3 in the main paper) across stages B to E (as per Table 2 in the main paper). Stage B (retrieve works) is initialized by search results. Stages C to E are initialized by taking the author, institution, and source IDs, respectively, as extracted from the works records retrieved in Stage B. Each step is facilitated by a suite of batch terminal files, as listed in the Figure below. Details for each step are provided in the remainder of this SM.

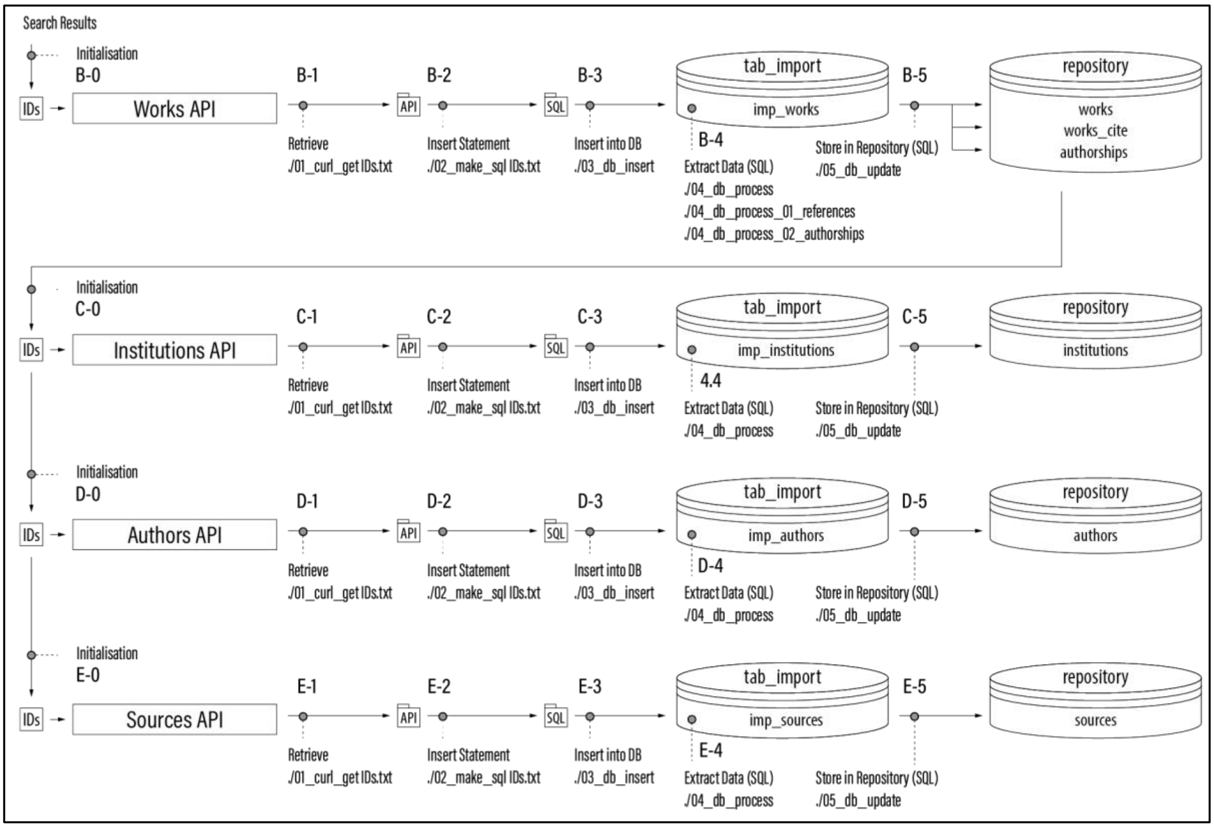

2 Retrieve Data from OpenAlex APIs

Step 1 uses MacOS terminal to retrieve individual records using OpenAlex APIs. The general structure of the CURL statements is as follows.

Works Retrieval API

Request URL

```
https://api.openalex.org/works/W1483146186?mailto=<emailAddress>
```

CURL with OpenAlexID

```
curl -X GET --header 'Accept: application/json'
'https://api.openalex.org/works/W1483146186?mailto=<emailAddress>'
```

CURL with pubmedID

```
curl -X GET --header 'Accept: application/json' 'https://api.openalex.org/works/
pmid:32203816?mailto=<emailAddress>'
```

CURL with doi

```
curl -X GET --header 'Accept: application/json' 'https://api.openalex.org/works/
https://doi.org/10.1080/10643389.2018.1558889?mailto=<emailAddress>'
```

Author Retrieval API

Request URL

```
https://api.openalex.org/authors/A2167930928? mailto=<emailAddress>
```

CURL with OpenAlexID

```
curl -X GET --header 'Accept: application/json' ' https://api.openalex.org/authors/
A2167930928?mailto=<emailAddress>'
```

Institution Retrieval API

Request URL

```
https://api.openalex.org/institutions/I298625061? mailto=<emailAddress>
```

CURL with OpenAlexID

```
curl -X GET --header 'Accept: application/json' ' https://api.openalex.org/institutions/
I298625061?mailto=<emailAddress>'
```

Sources Retrieval API

Request URL

```
https://api.openalex.org/sources/S169454776? mailto=<emailAddress>
```

CURL with OpenAlexID

```
curl -X GET --header 'Accept: application/json' ' https://api.openalex.org/sources/
S169454776?mailto=<emailAddress>'
```

2.1 General Structure

2.1.1 Bash Batch File

Opening

```
#!/bin/bash
```

Define eMail Address

```
email="<mail@server.net>"
```

Define and Select ID Types

```
<Specific Code Elements per API – see below>
```

Set Variables

```
x=0
y1=0
y2=0
z1=99
z2=999
```

Core

```
i=0
while IFS= read -r line || [[ -n "$line" ]]
do      ((i++))
        seq=$(printf "%06d" $i)
        <Specific Code Elements per API – see below>
        sleep 0.1
        if [[ $y1 -gt $z1 ]]
        then
        y1=0
        sleep 1
        fi
        if [[ $y2 -gt $z2 ]]
        then
        y2=0
        sleep 10
        fi
done < "$1"
```

2.1.2 Bash Execute Statement

```
./01_curl_get IDs.txt
```

2.2 Specific Code Elements per API

2.2.1 Works Retrieval API

Define and Select ID Types

```
PS2='Please choose type of identifier: '
options=("OAID" "DOI" "PMID")
select opt in "${options[@]}"
do
    case $opt in
        "OAID")
            identifier="OAID"
           echo "$identifier"
           break
            ;;
        <dito for DOI and PMID>
        *) echo "invalid option $REPLY";;
    esac
done
```

Core

```
if [[ $identifier == "OAID" ]]
then
echo "${seq} | OAID: ${line}"
curl -X GET --header 'Accept: application/json'
'https://api.openalex.org/works/'"$line"'?mailto='"$email" -o api/$seq.txt
elif [[ $identifier == "DOI" ]]
then
echo "${seq} | DOI: ${line}"
curl -X GET --header 'Accept: application/json'
'https://api.openalex.org/works/https://doi.org/'"$line"'?mailto='"$email" -o api/$seq.txt
<dito for PMID>
else
echo "ERROR"
fi
```

2.2.2 Authors Retrieval API

Define and Select ID Types

```
PS2='Please choose type of identifier: '
options=("OAID" "ORCID" "EID")
select opt in "${options[@]}"
do
    case $opt in
        "OAID")
            identifier="OAID"
           echo "$identifier"
           break
            ;;
        <dito for ORCID and EID>
        *) echo "invalid option $REPLY";;
    esac
done
```

Core

```
if [[ $identifier == "OAID" ]]
then
echo "${seq} | OAID: ${line}"
curl -X GET --header 'Accept: application/json'
'https://api.openalex.org/authors/'"$line"'?mailto=rharder@gmx.net' -o api/$seq.txt
elif [[ $identifier == "ORCID" ]]
then
echo "${seq} | ORCID: ${line}"
curl -X GET --header 'Accept: application/json'
'https://api.openalex.org/authors/https://orcid.org/'"$line"'?mailto=rharder@gmx.net' -o
api/$seq.txt
<dito for EID>
else
echo "ERROR"
fi
```

2.2.3 Institutions Retrieval API

Define and Select ID Types

```
PS2='Please choose type of identifier: '
options=("OAID" "ROR")
select opt in "${options[@]}"
do
    case $opt in
        "OAID")
            identifier="OAID"
           echo "$identifier"
           break
            ;;
        <dito for ROR>
        *) echo "invalid option $REPLY";;
    esac
done
```

Core

```
if [[ $identifier == "OAID" ]]
then
echo "${seq} | OAID: ${line}"
curl -X GET --header 'Accept: application/json'
'https://api.openalex.org/institutions/'"$line"'?mailto=rharder@gmx.net' -o api/$seq.txt
elif [[ $identifier == "ROR" ]]
then
echo "${seq} | ROR: ${line}"
curl -X GET --header 'Accept: application/json'
'https://api.openalex.org/institutions/https://ror.org/'"$line"'?mailto=rharder@gmx.net' -o
api/$seq.txt
else
echo "ERROR"
fi
```

2.2.4 Sources Retrieval API

Define and Select ID Types

```
PS2='Please choose type of identifier: '
options=("OAID" "ISSN")
select opt in "${options[@]}"
do
    case $opt in
        "OAID")
            identifier="OAID"
           echo "$identifier"
           break
            ;;
        <dito for ISSN>
        *) echo "invalid option $REPLY";;
    esac
done
```

Core

```
if [[ $identifier == "OAID" ]]
then
echo "${seq} | OAID: ${line}"
curl -X GET --header 'Accept: application/json'
'https://api.openalex.org/sources/'"$line"'?mailto=rharder@gmx.net' -o api/$seq.txt
elif [[ $identifier == "ISSN" ]]
then
echo "${seq} | ISSN: ${line}"
curl -X GET --header 'Accept: application/json'
'https://api.openalex.org/sources/issn:'"$line"'?mailto=rharder@gmx.net' -o api/$seq.txt
else
echo "ERROR"
fi
```

3 Load Records into Database Management System

Step 2 creates an SQL insert statement for each record that was retrieved through the respective OpenAlex API. Step 3 then executes the actual insertion into the database.

The general form of the insert statement is as follows.

```
INSERT IGNORE INTO <base_import_table> (sequence, query_key, query_val, query_result)
VALUES (<sequence>, <query_key>, <query_val>, <query_result>);
```

The base import tables per API are as follows.

API	Scheme	Table	Description
Works Retrieval	tab_import_api	openalex_api_works_imp	Base import table for works
Authors Retrieval	tab_import_api	openalex_api_authors_imp	Base import table for authors
Institutions Retrieval	tab_import_api	openalex_api_institutions_imp	Base import table for institutions
Sources Retrieval	tab_import_api	openalex_api_sources_imp	Base import table for sources

The variables are as follows.

Variable	Description	Examples
sequence	Sequence number in a list of IDs to be queried.	1,2, 3, etc.
query_key	Type of ID queried.	Work_id, author_id, affiliation_id, etc.
query_val	Value of the id queried	W1483146186, A2167930928, etc.
query_result	API response body	…

3.1 Create SQL Insert Statements – General Structure

3.1.1 Bash Batch File

Opening

```
#!/bin/bash
```

Define and Select ID Types

```
<Specific Code Elements per API – identical to Step 1b – see under 1.2 above>
```

Core

```
i=0
while IFS= read -r line || [[ -n "$line" ]]
do      ((i++))
        seq=$(printf "%06d" $i)
        <Specific per API – see below>
done < "$1"
```

3.1.2 Bash Execute Statement

```
./02_make_sql IDs.txt
```

IDs.txt Input File Structure

```
W1483146186
…
```

3.2 Create SQL Insert Statements – Specific Elements per API

3.2.1 Works Retrieval API

Core

```
if [[ $identifier == "OAID" ]]
        then
        echo "${seq} | OAID: ${line}"
        query_key="OAID"
        elif [[ $identifier == "DOI" ]]
        then
        echo "${seq} | DOI: ${line}"
        query_key="DOI"
        <dito for PMID>
        else
        echo "ERROR"
fi
> sql/sql_$seq.txt
echo "INSERT IGNORE INTO openalex_api_works_imp (sequence, query_key, query_val, query_result)
VALUES ('"$seq"', '"$query_key"', '"$line"', '" >> sql/sql_$seq.txt
cat api/$seq.txt | sed "s/\'/\\\'/g" >> sql/sql_$seq.txt
echo "'); " >> sql/sql_$seq.txt
```

3.2.2 Authors Retrieval API

Core

```
if [[ $identifier == "OAID" ]]
        then
        echo "${seq} | OAID: ${line}"
        query_key="OAID"
        elif [[ $identifier == "ORCID" ]]
        then
        echo "${seq} | ORCID: ${line}"
        query_key="ORCID"
        <dito for EID>
        else
        echo "ERROR"
fi
> sql/sql_$seq.txt
echo "INSERT IGNORE INTO openalex_api_authors_imp (sequence, query_key, query_val,
query_result) VALUES ('"$seq"', '"$query_key"', '"$line"', '" >> sql/sql_$seq.txt
cat api/$seq.txt | sed "s/\'/\\\'/g" >> sql/sql_$seq.txt
echo "'); " >> sql/sql_$seq.txt
```

3.2.3 Institutions Retrieval API

Core

```
if [[ $identifier == "OAID" ]]
        then
        echo "${seq} | OAID: ${line}"
        query_key="OAID"
        elif [[ $identifier == "ROR" ]]
        then
        echo "${seq} | ROR: ${line}"
        query_key="ROR"
        else
        echo "ERROR"
fi
> sql/sql_$seq.txt
echo "INSERT IGNORE INTO openalex_api_institutions_imp (sequence, query_key, query_val,
query_result) VALUES ('"$seq"', '"$query_key"', '"$line"', '" >> sql/sql_$seq.txt
cat api/$seq.txt | sed "s/\'/\\\'/g" >> sql/sql_$seq.txt
echo "'); " >> sql/sql_$seq.txt
```

3.2.4 Sources Retrieval API

Core

```
if [[ $identifier == "OAID" ]]
        then
        echo "${seq} | OAID: ${line}"
        query_key="OAID"
        elif [[ $identifier == "ISSN" ]]
        then
        echo "${seq} | ISSN: ${line}"
        query_key="ISSN"
        else
        echo "ERROR"
fi
> sql/sql_$seq.txt
echo "INSERT IGNORE INTO openalex_api_sources_imp (sequence, query_key, query_val,
query_result) VALUES ('"$seq"', '"$query_key"', '"$line"', '" >> sql/sql_$seq.txt
cat api/$seq.txt | sed "s/\'/\\\'/g" >> sql/sql_$seq.txt
echo "'); " >> sql/sql_$seq.txt
```

3.3 Import to Database Management System

3.3.1 Bash Batch File

Opening

```
#!/bin/bash
```

Core

```
export MYSQL_PWD=<password>

i=0
for filename in sql/*.txt; do
        ((i++))
        seq=$(printf "%06d" $i)
        xbase=${filename##*/}
        line=${xbase%.*}
        echo "$seq"
        mysql --host=localhost --user=repository tab_import_api < sql/$line.txt
done
```

3.3.2 Bash Execute Statement

```
./03_db_insert
```

4 Extract and Store Target Data

Step 4 extracts relevant bibliographic data elements from the recordsets previously retrieved from OpenAlex APIs and inserted to the database. Step 5 then is about storing the extracted data elements in a temporary local data repository. Data extraction takes place as detailed in the Figure below. Note that loops are required for target data elements with multiple elements. Multiple author keywords and concept terms are concatenated into one column in the respective auxiliary database table. Referenced and related works, as well as affiliations and authors are stored as separate records in the respective auxiliary database table.

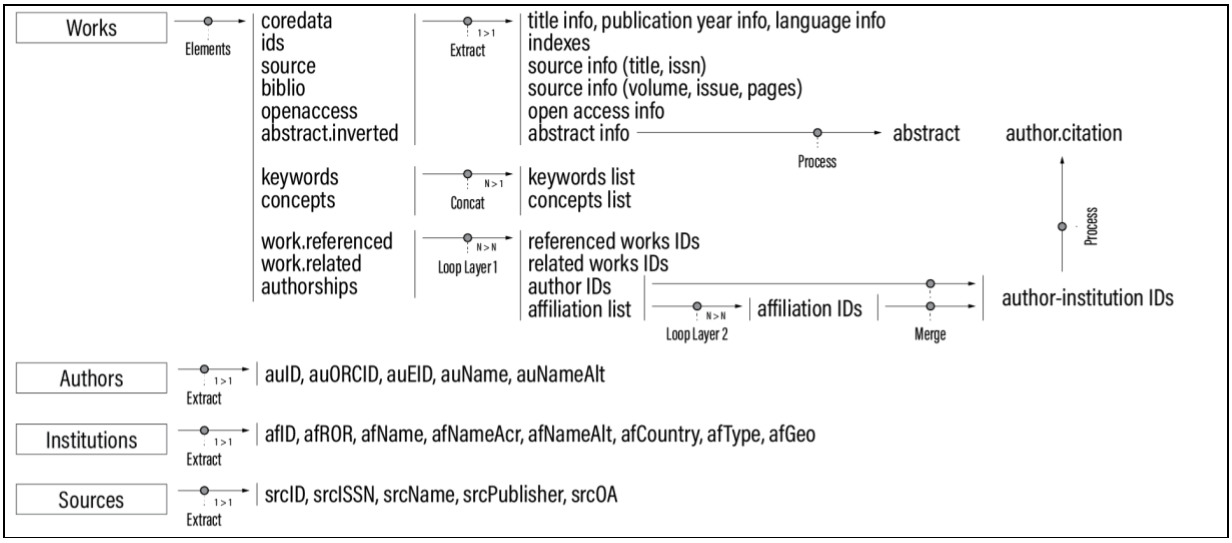

4.1 Data Extraction – Auxiliary Tables

In addition to the base import tables, the extraction of target bibliographic data requires a number of auxiliary tables, which are listed below per API.

4.1.1 Works Retrieval API

Scheme	Table	Description
tab_import_api	openalex_api_works_imp_val	Generic auxiliary table for processing various elements
tab_import_api	openalex_api_works_imp_ref	Auxiliary table for processing references
tab_import_api	openalex_api_works_imp_rel	Auxiliary table for processing related records
tab_import_api	openalex_api_works_imp_au_01	Auxiliary table for processing authors
tab_import_api	openalex_api_works_imp_au_02	Auxiliary table for processing authors
tab_import_api	openalex_api_works_imp_ab_01	Auxiliary table for processing abstracts
tab_import_api	openalex_api_works_imp_ab_02	Auxiliary table for processing abstracts
tab_import_api	openalex_api_works_imp_ab_03	Auxiliary table for processing abstracts
tab_import_api	openalex_api_works_imp_ab_04	Auxiliary table for processing abstracts
tab_import_api	openalex_api_works_imp_kw_xx	Auxiliary table for concatenating keywords into list

4.1.2 Author Retrieval API

Scheme	Table	Description
tab_import_api	---	No specific auxiliary table for processing authors

4.1.3 Institutions Retrieval API

Scheme	Table	Description
tab_import_api	---	No specific auxiliary table for processing institutions

4.1.4 Sources Retrieval API

Scheme	Table	Description
tab_import_api	---	No specific auxiliary table for processing sources

4.2 Data Extraction – Search Tags for Data Element Targeting

The extraction of target bibliographic data elements is done based on finding specific tags in the record. For all retrieval APIs, this relied on the JSON structure.

4.2.1 Works Retrieval API

Base Import Table

Column	#	Subset	Tag
oaid	1	query_result	"id":"https://openalex.org/ ... "
doi	1	query_result	"doi": " … "
json_ids	1	query_result	"ids":{ … }
json_source	1	query_result	"source":{ … }
json_biblio	1	query_result	"biblio":{ … }
json_openaccess	1	query_result	"open_access":{ … }
json_authorships	1	query_result	"authorships":[…]
json_keywords	1	query_result	"keywords":[{ … }]
json_concepts	1	query_result	concepts":[{ … }]
json_works_referenced	1	query_result	"referenced_works":[…]
json_works_related	1	query_result	"related_works":[…]
json_abstract_inverted_index	1	query_result	"abstract_inverted_index":{… }
title	1	query_result	"title":" … "
display_name	1	query_result	"display_name":" … "
publication_year	1	query_result	"publication_year":" … "
publication_date	1	query_result	"publication_date":" … "
language_iso2	1	query_result	"language":" … "
type_crossref	1	query_result	"type_crossref":" … "
is_retracted	1	query_result	"is_retracted": …
is_paratext	1	query_result	"is_paratext": …
ids_oax	1	json_ids	"openalex": …
ids_pmid	1	json_ids	"pmid": …
ids_pmcid	1	json_ids	"pmcid": …
ids_mag	1	json_ids	"mag": …
ids_doi	1	json_ids	"doi": …
src_id	1	json_source	"id":" …
src_display_name	1	json_source	"display_name":" … "
src_issn_l	1	json_source	"issn_l":[…]
src_issn	1	json_source	"issn":[…]
src_type	1	json_source	"type":" … "
bib_volume	1	json_biblio	"volume":" … "
bib_issue	1	json_biblio	"issue":" … "
bib_page_start	1	json_biblio	"first_page":" … "
bib_page_end	1	json_biblio	"last_page":" … "
oa_is_oa	1	json_openaccess	"is_oa":" … "
oa_status	1	json_openaccess	"oa_status":" … "
count_ref	1	json_works_referenced	# 'https://openalex.org/'
count_rel	1	json_works_related	# 'https://openalex.org/'
count_authors	1	json_authorships	# 'author_position'
abstract	1	json_abstract_inverted	Separate procedure
author_first	1	rec_api_works_imp_au_01	seq = 1 > author_displayname
author_second	1	rec_api_works_imp_au_01	seq = 2 > author_displayname
author_third	1	rec_api_works_imp_au_01	seq = 3 > author_displayname
citation_author	1		AU1 OR AU1 and AU 2 or AU1 et al.

Auxiliary Table for References [Loop Layer 1]

Column	#	Subset	Tag
oaid	1	oaid	
oaid_lnk	1	json_works_referenced	"https://openalex.org/ ... "

Auxiliary Table for Related Works [Loop Layer 1]

Column	#	Subset	Tag
oaid	1	oaid	
oaid_lnk	1	json_works_related	"https://openalex.org/ ... "

Auxiliary Table for Authorships [Loop Layer 1]

Column	#	Subset	Tag
oaid	1	oaid	
author	1	json_authorships	"author":
institutions	1	json_authorships	"institutions":
author_position	1	json_author	"author_position":
author_oaid	1	author	"id":"https://openalex.org/ ... "
author_displayname	1	author	"display_name":"
count_institutions	1	institutions	# "display_name":

Auxiliary Table for Authorships [Loop Layer 2]

Column	#	Subset	Tag
oaid	1	oaid	
auid	1	author_oaid	
afid	1	institutions	"id":"https://openalex.org/ ... "
auseq	1		author sequence per loop layer 1
afseq	1		author sequence per loop layer 2

4.2.2 *Authors Retrieval API*

Base Import Table

Column	#	Subset	Tag
oaid	1	query_result	"id":"https://openalex.org/ ... "
orcid	1	query_result	"orcid":"https://orcid.org/ ... "
scopus	1	query_result	"scopus": > authorID= … &
display_name	1	query_result	"display_name":" … "
display_name_alt	1	query_result	"display_name_alternatives":" … "

4.2.3 *Institutions Retrieval API*

Base Import Table

Column	#	Subset	Tag
oaid	1	query_result	"id":"https://openalex.org/ ... "
ror	1	query_result	"ror":"https://ror.org/ ... "
display_name	1	query_result	"display_name":" … "
display_name_acr	1	query_result	"display_name_acronyms":" … "
display_name_alt	1	query_result	"display_name_alternatives":" … "
institution_country	1	query_result	"country_code": …
institution_type	1	query_result	"type": …
geo	1	query_result	"geo": …
geo_city	1	geo	"city": …
geo_city_id	1	geo	"geonames_city_id": …
geo_region	1	geo	"region": …
geo_country_code	1	geo	"country_code": …
geo_country	1	geo	"country": …
geo_latitude	1	geo	"latitude": …
geo_longitude	1	geo	"longitude": …

4.2.4 *Sources Retrieval API*

Base Import Table

Column	#	Subset	Tag
oaid	1	query_result	"id":"https://openalex.org/ ... "
issn_l	1	query_result	"issn_l":" … "
issn	1	query_result	"issn":[…]
display_name	1	query_result	"display_name":" … "
publisher	1	query_result	"host_organization_name":" … "
is_oa	1	query_result	"is_oa": …

4.3 Data Extraction – Stored Procedures per API

Extracting target data elements and storing them in the local repository is facilitated by stored procedures, see below.

4.3.1 Works Retrieval API

Scheme	Stored Procedure	Description
tab_import_api	openalex_api_works_00_01_truncate	Truncate import tables
tab_import_api	openalex_api_works_01_extract_json_00_redirects	Identify redirects
tab_import_api	openalex_api_works_01_extract_json_01_remove_spaces	Remove spaces
tab_import_api	openalex_api_works_01_extract_json_02_split_json	Split json into multiple sub-fields
tab_import_api	openalex_api_works_02_extract_data_01_core	Extract core data
tab_import_api	openalex_api_works_02_extract_data_02_ids	Extract indexes
tab_import_api	openalex_api_works_02_extract_data_03_source	Extract source data
tab_import_api	openalex_api_works_02_extract_data_04_biblio	Extract bibliography data
tab_import_api	openalex_api_works_02_extract_data_05_openaccess	Extract openaccess data
tab_import_api	openalex_api_works_02_extract_data_06_abstract_xx	Convert abstract_inverted_index to abstract
tab_import_api	openalex_api_works_02_extract_data_07_keywords_xx	Concatenate keywords into list of keywords
tab_import_api	openalex_api_works_03_extract_ref_01	Count references and related works
tab_import_api	openalex_api_works_03_extract_ref_02	Extract references to auxiliary table [Loop Layer 1]
tab_import_api	openalex_api_works_03_extract_ref_03	Extract related works to auxiliary table [Loop Layer 1]
tab_import_api	openalex_api_works_03_extract_ref_04	Extract referring and referred OpenAlexIDs
tab_import_api	openalex_api_works_03_extract_ref_05	Extract relating and related OpenAlexIDs
tab_import_api	openalex_api_works_04_extract_authorships_01	Count authors
tab_import_api	openalex_api_works_04_extract_authorships_02	Extract authorship data
tab_import_api	openalex_api_works_04_extract_authorships_03_01	Extract author and institution data [Loop Layer 1]
tab_import_api	openalex_api_works_04_extract_authorships_03_02	Extract author data elements
tab_import_api	openalex_api_works_04_extract_authorships_04	Count institutions per author
tab_import_api	openalex_api_works_04_extract_authorships_05_01	Extract institutions data [Loop Layer 2]
tab_import_api	openalex_api_works_04_extract_authorships_05_02	Extract institutions data elements
tab_import_api	openalex_api_works_04_extract_authorships_06	Extract authorship data elements
tab_import_api	openalex_api_works_04_extract_authorships_07	Create author date identifier

4.3.2 Authors Retrieval API

Scheme	Stored Procedure	Description
tab_import_api	openalex_api _authors_00_01_truncate	Truncate import tables
tab_import_api	openalex_api _authors_01_extract_data_01_core	Extract core data

4.3.3 Institutions Retrieval API

Scheme	Stored Procedure	Description
tab_import_api	openalex_api_institutions_00_01_truncate	Truncate import tables
tab_import_api	openalex_api_institutions_01_extract_data_01_core	Extract core data
tab_import_api	openalex_api_institutions_01_extract_data_02_geo	Extract geo data

4.3.4 Sources Retrieval API

Scheme	Stored Procedure	Description
tab_import_api	openalex_api_sources_00_01_truncate	Truncate import tables
tab_import_api	openalex_api_sources_01_extract_data_01_core	Extract core data

4.4 Data Storage – Local Repository Tables

For storage of target bibliographic data elements in a local temporary repository, a number of tables were used, see below.

4.4.1 Works Retrieval API

Scheme	Table	Description
repository_api_openalex	rec_api_works	Table for extracted bibliographic data
repository_api_openalex	rec_api_authorships	Table for extracted authorships
repository_api_openalex	rec_api_works_cite_ref	Table for extracted references
repository_api_openalex	rec_api_works_cite_rel	Table for extracted related works
repository_api_openalex	rec_api_works_idx_doi	Table for extracted doi
repository_api_openalex	rec_api_works_idx_pmid	Table for extracted pmid
repository_api_openalex	dbs_api_works_alias	Table for aliases
repository_api_openalex	dbs_api_works_data_records	Table for raw records
repository_api_openalex	dbs_api_works_data_records_errors	Table for errors

4.4.2 Authors Retrieval API

Scheme	Table	Description
repository_api_openalex	rec_api_authors	Table for extracted bibliographic data
repository_api_openalex	dbs_api_authors_alias	Table for aliases
repository_api_openalex	dbs_api_authors_data_records	Table for raw records
repository_api_openalex	dbs_api_authors_data_records_errors	Table for errors

4.4.3 Institutions Retrieval API

Scheme	Table	Description
repository_api_openalex	rec_api_institutions	Table for extracted bibliographic data
repository_api_openalex	dbs_api_institutions_alias	Table for aliases
repository_api_openalex	dbs_api_institutions_data_records	Table for raw records
repository_api_openalex	dbs_api_institutions_data_records_errors	Table for errors

4.4.4 Sources Retrieval API

Scheme	Table	Description
repository_api_openalex	rec_api_sources	Table for extracted bibliographic data
repository_api_openalex	dbs_api_sources_alias	Table for aliases
repository_api_openalex	dbs_api_sources_data_records	Table for raw records
repository_api_openalex	dbs_api_sources_data_records_errors	Table for errors

4.5 Data Storage – Stored Procedures

The storage of target bibliographic data elements in a local temporary repository was facilitated by stored procedures, see below.

4.5.1 Works Retrieval API

Scheme	Stored Procedure	Description
tab_import_api	openalex_api_works_05_populate_01_works_data	Populate table for core data
tab_import_api	openalex_api_works_05_populate_02_works_data_records	Populate table for raw records
tab_import_api	openalex_api_works_05_populate_03_works_data_records_errors	Populate table for errors
tab_import_api	openalex_api_works_05_populate_04_works_ref	Populate table for references
tab_import_api	openalex_api_works_05_populate_05_works_rel	Populate table for related works
tab_import_api	openalex_api_works_05_populate_06_works_authorships	Populate table for authorships
tab_import_api	openalex_api_works_05_populate_07_works_doi	Populate table for doi
tab_import_api	openalex_api_works_05_populate_07_works_pmid	Populate table for pmid

4.5.2 Authors Retrieval API

Scheme	Stored Procedure	Description
tab_import_api	openalex_api_authors_02_populate_01_authors_data	Populate table for core data
tab_import_api	openalex_api_authors_02_populate_02_authors_data_records	Populate table for raw records
tab_import_api	openalex_api_authors_02_populate_03_authors_data_records_errors	Populate table for errors
tab_import_api	openalex_api_authors_02_populate_04_authors_alias	Populate table for Aliases

4.5.3 Institutions Retrieval API

Scheme	Stored Procedure	Description
tab_import_api	openalex_api_institutions_02_populate_01_institutions_data	Populate table for core data
tab_import_api	openalex_api_institutions_02_populate_02_institutions_data_records	Populate table for raw records
tab_import_api	openalex_api_institutions_02_populate_03_institutions_data_records_errors	Populate table for errors
tab_import_api	openalex_api_institutions_02_populate_04_institutions_alias	Populate table for Aliases

4.5.4 Sources Retrieval API

Scheme	Stored Procedure	Description
tab_import_api	openalex_api_sources_02_populate_01_sources_data	Populate table for core data
tab_import_api	openalex_api_sources_02_populate_02_sources_data_records	Populate table for raw records
tab_import_api	openalex_api_sources_02_populate_03_sources_data_records_errors	Populate table for errors
tab_import_api	openalex_api_sources_02_populate_04_sources_alias	Populate table for Aliases

4.6 Bash Files per API

To facilitate smooth and automated calling of the stored procedures, a number of bash batch files were used, see below.

4.6.1 Works Retrieval API

Folder	Bash File	Description
OPENALEX_WORKS	04_db_process	Extract core elements
OPENALEX_WORKS	04_db_process_00_abstracts	Extract abstracts
OPENALEX_WORKS	04_db_process_01_references	Extract references
OPENALEX_WORKS	04_db_process_02_authorships	Extract authors and institutions
OPENALEX_WORKS	05_db_update	Update local temporary repository

4.6.2 Authors Retrieval API

Folder	Bash File	Description
OPENALEX_AUTHORS	04_db_process	Extract core elements
OPENALEX_AUTHORS	05_db_update	Update local temporary repository

4.6.3 Institutions Retrieval API

Folder	Bash File	Description
OPENALEX_INSTITUTIONS	04_db_process	Extract core elements
OPENALEX_INSTITUTIONS	05_db_update	Update local temporary repository

4.6.4 Sources Retrieval API

Folder	Bash File	Description
OPENALEX_SOURCES	04_db_process	Extract core elements
OPENALEX_SOURCES	05_db_update	Update local temporary repository
